# A gradient of frequency-dependent synaptic properties along the longitudinal hippocampal axis

**DOI:** 10.1186/s12868-017-0398-4

**Published:** 2017-12-12

**Authors:** Vassilios Papaleonidopoulos, George Trompoukis, Andriana Koutsoumpa, Costas Papatheodoropoulos

**Affiliations:** Department of Medicine, Laboratory of Physiology, University of Patras, 26504 Rion, Greece

**Keywords:** Hippocampus, Dorsoventral, Septotemporal, Short-term plasticity, Facilitation, Depression, Frequency stimulation, Theta rhythm, Beta rhythm, In vitro

## Abstract

**Background:**

The hippocampus is a functionally heterogeneous brain structure and specializations of the intrinsic neuronal network may crucially support the functional segregation along the longitudinal axis of the hippocampus. Short-term synaptic plasticity plays fundamental roles in information processing and may be importantly involved in diversifying the properties of local neuronal network along the hippocampus long axis. Therefore, we aimed to examine the properties of the cornu ammonis 1 (CA1) synapses along the entire dorsoventral axis of the rat hippocampus using field excitatory postsynaptic potentials from transverse rat hippocampal slices and a frequency stimulation paradigm.

**Results:**

Applying a ten-pulse stimulus train at frequencies from 0.1 to 100 Hz to the Schaffer collaterals we found a gradually diversified pattern of frequency-dependent synaptic effects along the dorsoventral hippocampus axis. The first conditioned response was facilitated along the whole hippocampus for stimulus frequencies 10–40 Hz. However, steady-state responses or averaged responses generally ranged from maximum synaptic facilitation in the most dorsal segment of the hippocampus to maximum synaptic depression in the most ventral segment of the hippocampus. In particular, dorsal synapses facilitated for stimulus frequency up to 50 Hz while they depressed at higher frequencies (75–100 Hz). Facilitation at dorsal synapses was maximal at stimulus frequency of 20 Hz. On the contrary, the most ventral synapses showed depression regardless of the stimulus frequency, only displaying a transient facilitation at the beginning of 10–50 Hz stimulation. Importantly, the synapses in the medial hippocampus displayed a transitory behavior. Finally, as a whole the hippocampal synapses maximally facilitated at 20 Hz and increasingly depressed at 50–100 Hz.

**Conclusion:**

The short-term synaptic dynamics change gradually along the hippocampal long axis in a frequency-dependent fashion conveying distinct properties of information processing to successive segments of the structure, thereby crucially supporting functional segregation along the dorsoventral axis of the hippocampus.

## Background

The hippocampus is an elongated functionally heterogeneous brain structure in which different functions have been ascribed to discrete segments along its longitudinal axis [1–3]. The mechanisms of this functional segregation along the long axis of the hippocampus (called also septotemporal or dorsoventral axis) are largely unknown, though the distinct patterns of extrinsic anatomical connections appear to play a significant role [4, 5]. Moreover, it should be expected that specializations in the computations performed by the endogenous hippocampal circuitry may also occur along its dorsoventral extent significantly participating in the segregation of functions along the dorsoventral hippocampal axis. Indeed, a recently growing body of evidence shows that a plethora of different aspects in the organization of the intrinsic hippocampal neural network are markedly diversified along the long axis of the structure [3, 6] despite the fact that general characteristics of the basic circuitry are kept similar along the long hippocampus axis [7, 8]. The knowledge therefore of the seemingly extensive repertoire of specializations that occur along the longitudinal axis of the hippocampus may crucially contribute to our understanding of the mechanisms underlying functional segregation along the hippocampus.

Synaptic plasticity, the ability of synapses to undergo lasting changes in their effectiveness [9, 10] is thought to play fundamental roles in brain functions and behavior [11, 12] and is importantly involved in information processing in the hippocampus [13]. Therefore, synaptic plasticity can be decisively involved in diversifying the intrinsic neural computations performed by different segments along the hippocampus long axis. Indeed, the ability of synapses for long-term potentiation, the most accepted mechanism assumed to underlie learning and memory [14–16] is conspicuously different between the dorsal and the ventral hippocampal synapses [17–20]. In addition to long-term plasticity, hippocampal synapses display a large variety of short-term plasticity phenomena [21], which constitute transient forms of activity-dependent variations of the synaptic efficacy [22–24].

Short-term synaptic plasticity determines the content of information transmitted between neurons thereby playing fundamental roles in information processing by cortical networks [25, 26]. For instance, cortical synapses can act as filters between presynaptic and postsynaptic activity permitting the flow of information contained in a presynaptic spike train of a particular frequency [26, 27]. Furthermore, evidence shows that short-term synaptic plasticity importantly contributes to controlling the flow of activity in the transversal axis of the hippocampus (i.e. through the trisynaptic circuit) [28, 29], and the frequency of hippocampal activation is a critical factor determining the extent of activity propagation from the hippocampus to cortical and subcortical structures [29]. Also, synapses can work as gain controllers thereby keeping the dynamic fidelity of input information [30, 31]. Therefore, the various forms of short-term plasticity may significantly participate to diversifying local neuronal circuitry along the hippocampus long axis.

Individual hippocampal synapses exhibit considerable variability in their properties [32–34], although they are usually perceived as having almost identical characteristics as a group. Recent evidence, however, has shown that large population of synapses in the dorsal and the ventral segment of the hippocampus display markedly different properties as for instance paired-pulse facilitation [20, 35–38] and frequency facilitation [39]. This has been suggested to arise from considerable differences in intrinsic synaptic properties such as the probability of transmitter release [35, 37]. Analyzing therefore the dynamics of short-term synaptic plasticity and understanding the computational processes operated by the local circuitry along the longitudinal axis of the hippocampus is of major importance for understanding how hippocampus achieves its diverse functions. Motivated by the essential involvement of short-term plasticity in neural network functioning and the fact that the frequency of activation is a basic parameter that determines the direction and the amount of plastic synaptic changes [23, 26, 40–42] we sought to determine the properties of short-term synaptic plasticity along the entire longitudinal axis of the rat hippocampus using field recordings from transverse hippocampal slices and a frequency stimulation paradigm. We show that short-term synaptic dynamics are remarkably and gradually diversified along the longitudinal hippocampal axis.

## Methods

### Hippocampal slice preparation

**Fig. 1:**
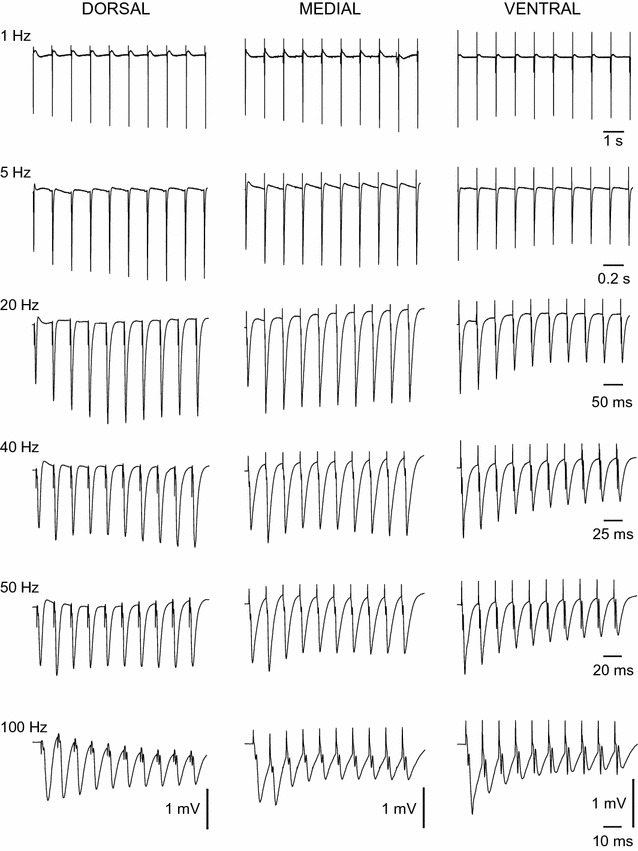
Examples of synaptic responses (fEPSPs) evoked by a stimulation train of ten pulses delivered at different representative frequencies are shown for three representative slice positions along the dorsoventral axis of the hippocampus. Records were obtained from three slices located 1, 6 and 9.5 mm along the dorsoventral axis of the hippocampus, corresponding to the most dorsal (DORSAL), medial (MEDIAL) and most ventral part (VENTRAL) of the hippocampus. Baseline fEPSPs in the three slices had similar amplitude (~ 1.5 mV). Note that the steady-state response in the dorsal slice was facilitation for most stimulation frequencies (1–40 Hz) and only at 100 Hz showed a moderate depression. In sharp contrast, the response of the ventral hippocampal slice was depression for all stimulus frequencies. The responses evoked in the medial hippocampus slice were intermediate between those recorded from the dorsal and the ventral slice

Adult Wistar male rats (2–3 month-old) used in this study were maintained at the Laboratory of Experimental Animals of the Department of Medicine, University of Patras. Experiments were conducted in accordance with the European Communities Council Directive Guidelines for the care and use of Laboratory animals (2010/63/EU—European Commission) and they have been approved by the “Protocol Evaluation Committee” of the Department of Medicine of the University of Patras and the Directorate of Veterinary Services of the Achaia Prefecture of Western Greece Region (# EL-13-BIOexp-04). In addition, all efforts have been made to minimize the number of animals used as well as their suffering. Rats were maintained under stable conditions of light–dark cycle (12/12 h), temperature (20–22 °C) and they had free access to food and water. In each experiment an animal was decapitated under deep anaesthesia with diethyl-ether, the brain was removed from the cranium and placed in ice-cold (2–4 °C) standard artificial cerebrospinal fluid (ACSF) containing, in mM: 124 NaCl, 4 KCl, 2 CaCl_2_, 2 MgSO_4_, 26 NaHCO_3_, 1.25 NaH_2_PO_4_ and 10 glucose. ACSF was equilibrated with 95% O_2_ and 5% CO_2_ gas mixture at a pH = 7.4. Under these conditions the hippocampus was excised free and placed on the disc of a McIlwain tissue chopper. Transverse 500–550 µm-thick slices were prepared from the whole hippocampus as previously described [37]. In order to maintain an orthogonal cut plane across the whole longitudinal axis of the hippocampus a slight ~ 10°–15° turns of the plate supporting the structure in the chopper were required to be made when sectioning medial positions of the hippocampus. As can be seen in Fig. 1, the hippocampus can be divided into about twenty slices since the longitudinal (septo-temporal or dorso-ventral) dimension of the adult rat hippocampus measures approximately 11 mm. Therefore, we allocated twenty *slice positions* along the longitudinal axis of the hippocampus, i.e. from 1st, at the dorsal end to the 20th at the ventral hippocampus end. However, the slices obtained from the most extreme positions (1st and 20th) did not usually give reliable responses and slices from these positions were excluded from the study. Therefore, in this study we used a maximum of eighteen transverse slices from a hippocampus. Immediately after sectioning, slices were transferred to an interface type recording chamber where they were maintained for later experimentation continuously perfused with ACSF at a rate of ~ 1.5 ml/min and humidified with a mixed gas consisting of 95% O2 and 5% CO2 at a constant temperature of 31 ± 0.5 °C. Tissue stimulation and recording started at least one and a half hours after their placement in the chamber.

### Recordings, data processing and analysis

Field excitatory postsynaptic potentials (fEPSPs) were recorded from the middle stratum radiatum of the CA1 field following electrical stimulation of the Schaffer collaterals. fEPSP was seen as a negative deflection away from the baseline (see Fig. 1). A wire-made bipolar platinum/iridium electrode with a wire diameter of 25 μm and an inter-wire distance of 100 μm (World Precision Instruments, USA) was used for electrical stimulation and placed toward the cornu ammonis 3 (CA3) field. Electrical pulses had a fixed duration of 100 μs and amplitude that varied from 20 to 250 μA. Recordings were made using a 7 μm-thick carbon fiber (Kation Scientific, Minneapolis, USA) positioned 350 μm from the stimulation electrode. Baseline stimulation was delivered every 30 s using a current intensity that elicited a just-subthreshold fEPSP on the basis of input/output curves between stimulation intensity and evoked responses. However, consecutive trains of pulses, delivered during the application of frequency stimulation paradigm, were separated by a two-minute interval in order to allow synapses to return to baseline level. Signal was amplified 500 times and band-pass filtered at 0.5 Hz–2 kHz using a Neurolog amplifier (Digitimer Limited, UK), digitized at 10 kHz and stored on a computer disk for off-line analysis using the CED 1401-plus interface and the Signal6 software (Cambridge Electronic Design, Cambridge, UK). Only slices which displayed stable response for about 10 min were selected for further experimentation. In order to examine the frequency-dependent properties of the CA1 synapses, the experimental paradigm of frequency stimulation was employed, which consisted of a train of ten pulses delivered at varying frequencies from 0.1 to 100 Hz. In particular, the frequencies of 0.1, 1, 3, 5, 10, 20, 30, 40, 50, 75 and 100 Hz were used. The number of pulses falls into the range of naturally occurring spike trains in CA3 cells [43]. The effects of frequency stimulation were quantified as the percent change of each of the nine consecutive fEPSPs evoked by the consecutive pulses with respect to the first fEPSP in the train. The frequency stimulation paradigm was applied at the baseline stimulation current intensity. fEPSP was quantified by the maximum slope of its rising phase. The nonparametric Wilcoxon Signed Rank test and the Friedman Two-Way Analysis of Variance (ANOVA) test were used for statistical comparisons. The values of the various parameters are expressed as mean ± S.E.M.

## Results

### Short-term synaptic plasticity along the whole hippocampus dorsoventral axis

**Fig. 2:**
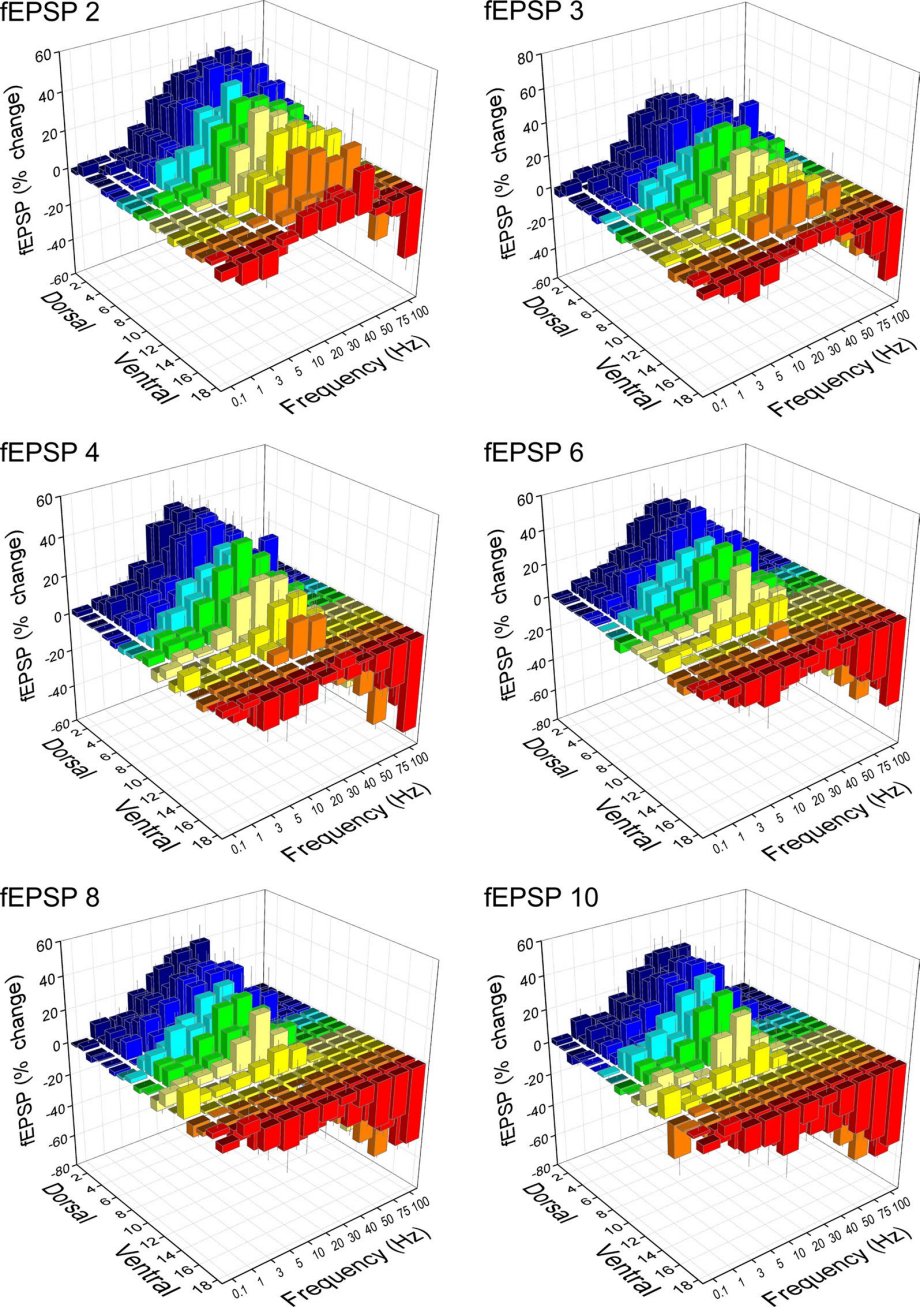
Stimulation-induced short-term changes in synaptic transmission plotted as a function of stimulus frequency and the slice position along the dorsoventral axis of the hippocampus. Results for six representative fEPSPs evoked during the ten-pulse stimulation train are presented in the different 3D graphs. In order to better illustrate fEPSP changes induced by low and high stimulation frequencies the left and right views of the 3D graphs are shown in separate figures. In particular, the left view is shown here whereas the right view is presented in the following Fig. 3. Note that the most dorsal hippocampal slices displayed facilitation over a wide range of stimulation frequencies (from 1 Hz to 40 Hz) and over the whole stimulation train whereas the most ventral slices showed depression over the entire range of stimulation frequencies and the duration of stimulation. Also, note that the response changed from facilitation to depression gradually rather than abruptly from the dorsal to the ventral hippocampus

**Fig. 3:**
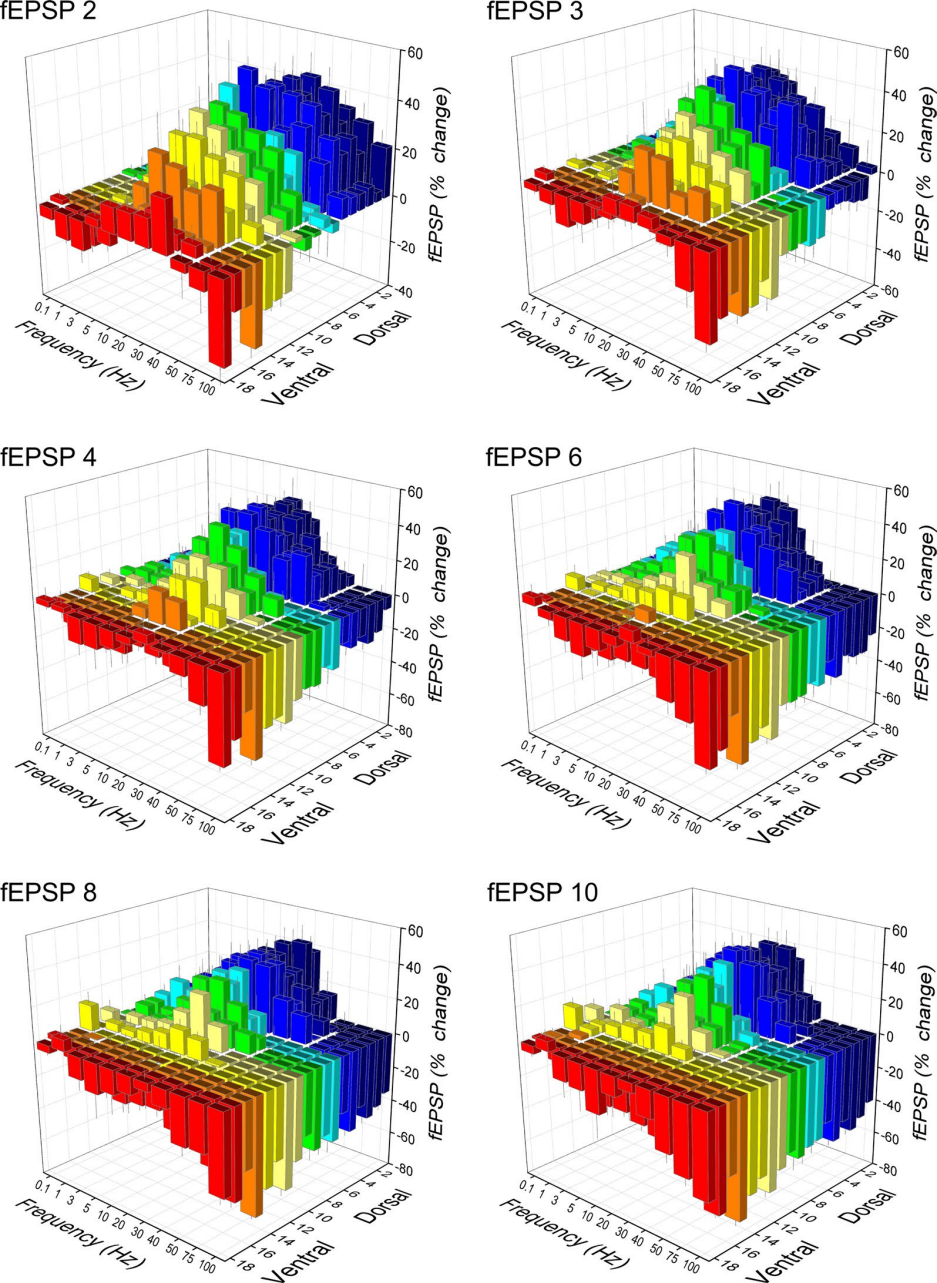
Stimulation-induced short-term changes in synaptic transmission plotted as a function of stimulus frequency and the slice position along the dorsoventral axis of the hippocampus. Here are presented the right views of 3D graphs shown in Fig. 2

**Fig. 4:**
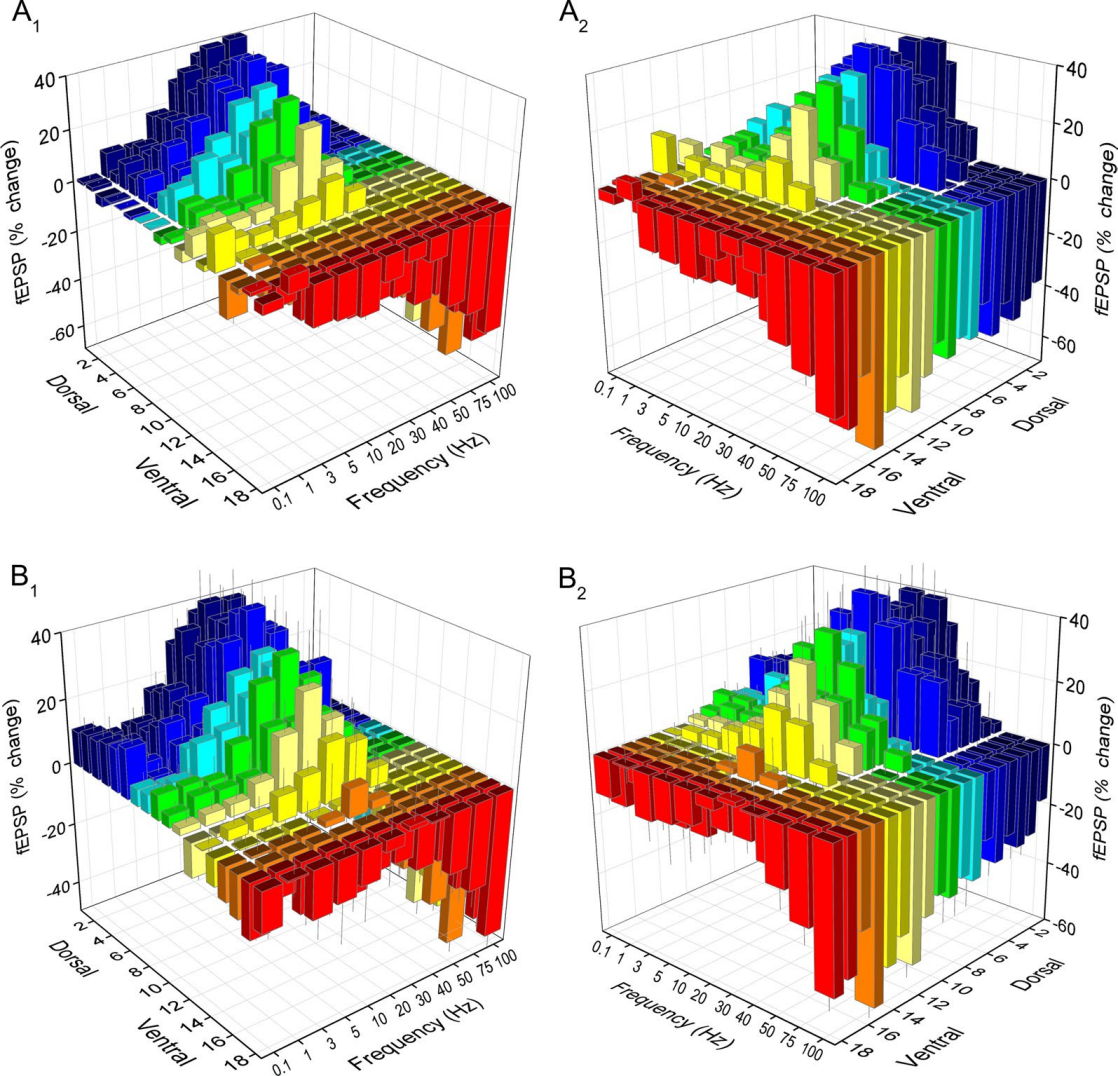
Stimulus-induced changes in fEPSP plotted against slice position and stimulus frequency. The average change of 8th-to-10th responses and of all conditioned (i.e. 2nd-to-10th) responses are shown in A and B respectively. The left and right views of the 3D graphs are shown separately in the left (A_1_, B_1_) and right plots (A_2_, B_2_)

We recorded fEPSPs from one hundred seventeen slices prepared from the hippocampi of ten rats. The number of slices obtained from eighteen consecutive positions along the dorsoventral axis of the hippocampus and the corresponding number of rats are given in Table 1. The stimulation paradigm of a ten-pulse train was applied at a stimulation current intensity that evoked a just-subthreshold fEPSP, which had a mean slope of 0.72 ± 0.33 mV/ms (n = 117). Examples of the effects of frequency stimulation observed at selected frequencies and slice positions along the dorsoventral axis of the hippocampus are presented in Fig. 1. Figures 2 and 3 shows that the frequency stimulation paradigm induced remarkably different frequency-dependent effects in slices obtained from different positions along the dorsoventral axis of the hippocampus. In general, stimulation applied at frequencies 1–40 Hz induced synaptic facilitation that was higher at the dorsal segment of the hippocampus and declined gradually along the dorsoventral axis eventually turning into synaptic depression at most ventral parts; stimulation at 0.1 Hz did not produce any significant change in synaptic transmission while the average response at frequencies 50–100 Hz was depression that increased along the dorsoventral axis. Nevertheless, some deviation was seen for the 2nd response in the train, which at the frequency range of 5–40 Hz facilitated along the whole hippocampal axis. In particular, the facilitation of the 2nd response was highest at more dorsal positions and then declined gradually and significantly toward the ventral hippocampus (ANOVA for each frequency between 5 and 40 Hz, 1.8 ≤ F ≤ 2.4, *P* < 0.05). At stimulation frequencies 1–3 Hz the more dorsal segment of the hippocampus showed only a negligible facilitation (1.2 ± 1.2 and 3.9 ± 1.4% respectively, Mann–Whitney U test for positions 2-6, *P* > 0.05) while in the most ventral segment (positions 15-19) a moderate but significant depression was observed (− 5.9 ± 2.0% and − 7.4 ± 3.5% for 1 and 3 Hz respectively, Mann–Whitney U test, *P* < 0.05). At high stimulation frequencies of 50–100 Hz a gradual change from facilitation to depression was observed along the dorsoventral axis (ANOVA, F = 2.9, *P* < 0.001). Actually, the facilitation of the 2nd response corresponds to paired-pulse facilitation, which has been previously found to be higher in dorsal than in ventral CA1 synapses at inter-pulse intervals ≥ 20 ms (corresponding to ≤ 50 Hz) [35, 37, 38, 44]. The following responses in the train (i.e. 3–10) showed a gradual transition from facilitation to depression when increasing stimulus frequency and from dorsal to ventral positions. Thus, with increased number of pulses the facilitation was restricted to dorsal and to medial hippocampal positions and to frequencies up to 40–50 Hz. At higher frequencies even dorsal synapses showed steady-state depression. Strikingly, the ventral synapses (positions > 15) consistently depressed over the entire frequency range (Fig. 3). When steady-state responses (i.e. from 8th to 10th) were averaged and plotted as a function of stimulus function and slice position along the dorsoventral axis of the hippocampus (Fig. 4a) we found that for stimulus frequencies from 1 to 40 Hz there was a gradual transition from facilitation in the dorsal segment to depression in the ventral segment (ANOVA along the whole axis, 4.5 ≤ F ≤ 14.8, *P* < 0.001). Notably, the facilitation in dorsal synapses (positions 2–6) and at frequencies 5–30 Hz remained strong over the entire stimulation train. At 50 Hz dorsal synapses did not show any appreciable change while ventral synapses depressed (ANOVA along the axis, F = 3.4, *P* < 0.001). For stimulus frequencies of 75–100 Hz there was a gradually increasing depression along the dorsoventral hippocampus axis (ANOVA along the axis, 2.9 ≤ F ≤ 3.3, *P* < 0.001). At 0.1 Hz we did not observe any consistent pattern of changes along the dorsoventral axis. We observed a similar pattern of changes when all conditioned responses (i.e. from 2nd to 10th) were averaged and plotted against stimulus frequency and slice position (Fig. 4b), but the gradual transition from facilitation to depression along the dorsoventral axis was seen also at the lowest stimulus frequency of 0.1 Hz.

**Table 1:** Number of slices and animals used from 18 positions along the dorsoventral axis of the hippocampus

Segment	Dorsal						Medial							Ventral				
Position	2	3	4	5	6	7	8	9	10	11	12	13	14	15	16	17	18	19
Slices	8	10	9	6	8	7	5	7	6	7	6	7	4	4	6	5	8	4
Rats	7	9	8	5	7	5	5	6	6	6	5	5	3	4	6	5	7	3

**Fig. 5:**
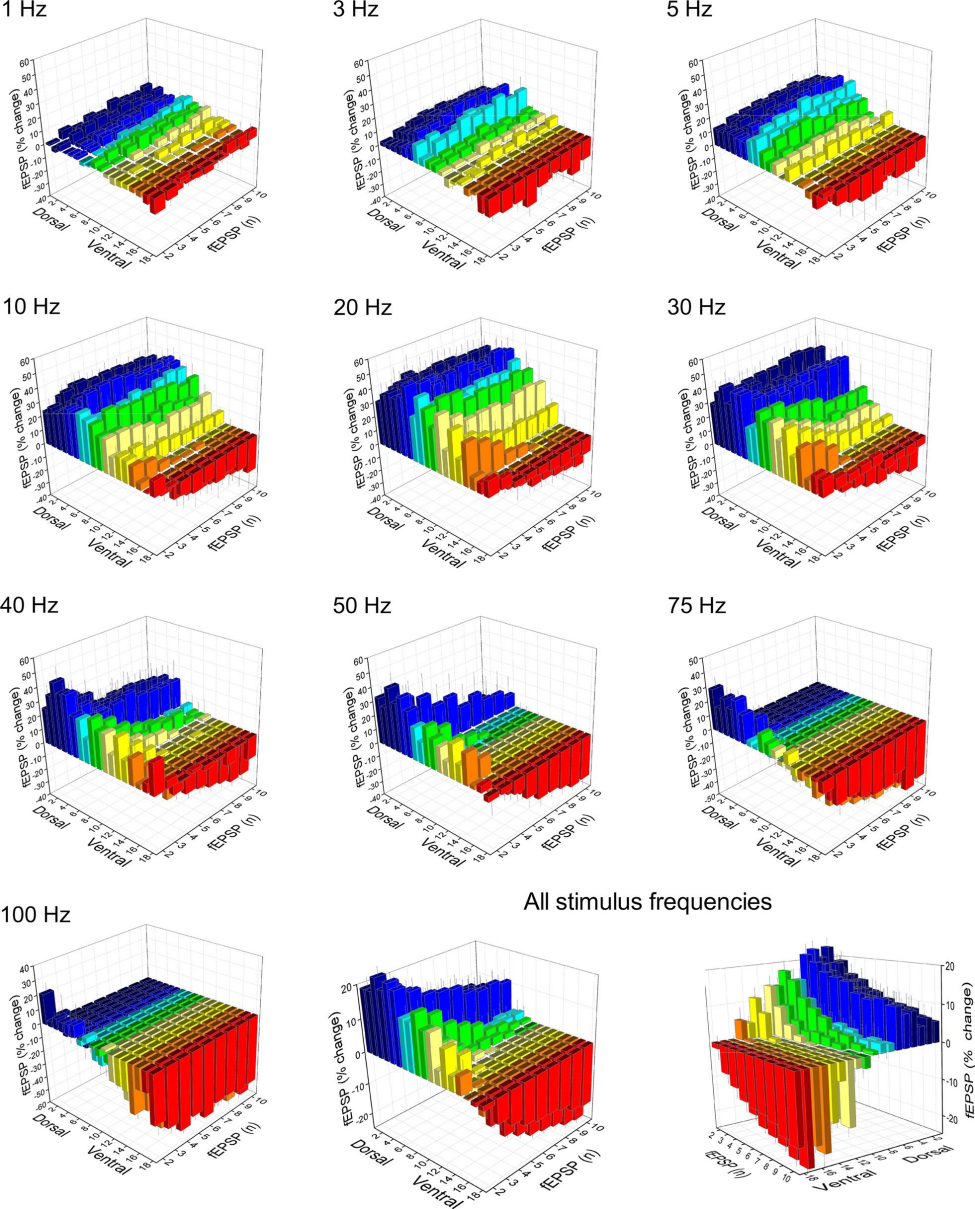
Frequency-dependent changes in synaptic transmission plotted as a function of slice position in the dorsoventral axis and the number of evoked fEPSP along the stimulus train. Data obtained at different stimulus frequencies are presented in ten different 3D graphs, indicated accordingly. The average results of all stimulation frequencies are presented in the last two graphs (left and right views) at the bottom right corner of the figure. Note that the synaptic facilitation starts at low stimulus frequency (1 Hz), progressively increases in the dorsal hippocampus, maximizes at 20 Hz and gradually declines toward the ventral hippocampus and at higher frequencies. Also note that regardless of the stimulus frequency there is a gradual change in synaptic responses from increased facilitation at the beginning of stimulation in dorsal hippocampal synapses to increased depression in the ventral synapses as stimulation progressed, while the synapses in the medial hippocampus showed a transitory behavior

In order to better illustrate the effects of repetitive activation on the synaptic responses over the ten-pulse train we plotted the short-term synaptic changes separately for the various stimulus frequencies (Fig. 5). As can be seen, at frequencies 1 to 5 Hz, facilitation was the typical response for synapses located at positions 2–14 along the dorsoventral axis (dorsal and medial hippocampal synapses), while synapses located at positions 15–19 (ventral hippocampal synapses) steadily depressed. At 10–30 Hz we observed that from the medial to ventral hippocampus (positions 8–16) the facilitation of the first response declined during repetitive activation. At 50 Hz, dorsal synapses (positions 2–7) facilitated over the entire stimulus train while medial hippocampus synapses facilitated only at the beginning of stimulation (i.e. during the first 2–3 responses). At higher frequencies, facilitation gradually fainted during the first responses and from dorsal to medial hippocampal positions. Computing the average response of all stimulus frequencies for each conditioned response we observed that maximum facilitation occurred at synapses of the most dorsal segment of the hippocampus extended 3.5–4.0 mm from the dorsal end of the structure (positions 2–7) and at the beginning of the stimulus train. Facilitation progressively declined along the medial part of the hippocampus (positions 8–14) and during the stimulus train and was replaced by depression that increased as the stimulus progressed and maximized at more ventral positions of the hippocampus.

### Short-term synaptic plasticity in dorsal, medial and ventral hippocampus

**Fig. 6:**
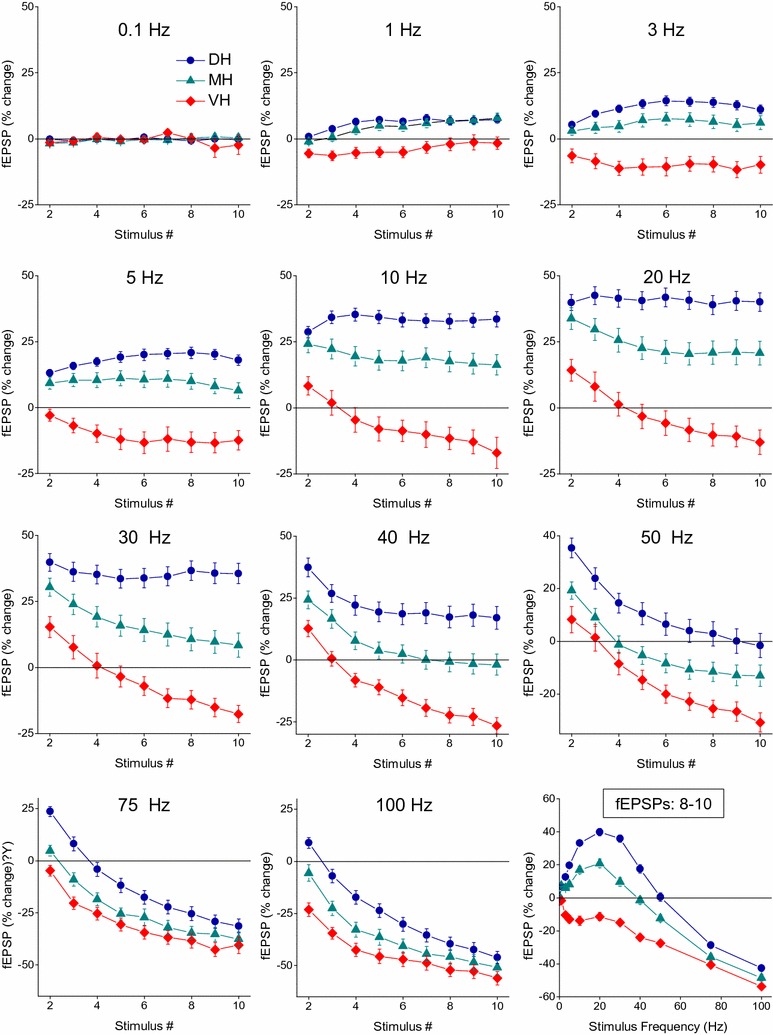
Synaptic responses induced by the ten-pulse stimulation train at the dorsal, medial and ventral hippocampal synapses plotted against stimulus number. Different diagrams illustrate the responses induced by the eleven different stimulation frequencies. The graph at the bottom right corner of the figure illustrate the steady-state responses (8–10) of the three hippocampal segments plotted as a function of stimulus frequency. Note that facilitation dominates dorsal hippocampal synapses at stimulus frequencies 1–50 Hz whereas ventral hippocampal synapses are dominated by depression at all stimulus frequencies showing only a transient facilitation at the start of 10–50 Hz stimulation. The synapses of the medial hippocampus show intermediate properties. Data for the dorsal, medial and ventral hippocampus were obtained from positions 2–7, 8–14 and 15–19 respectively

**Fig. 7:**
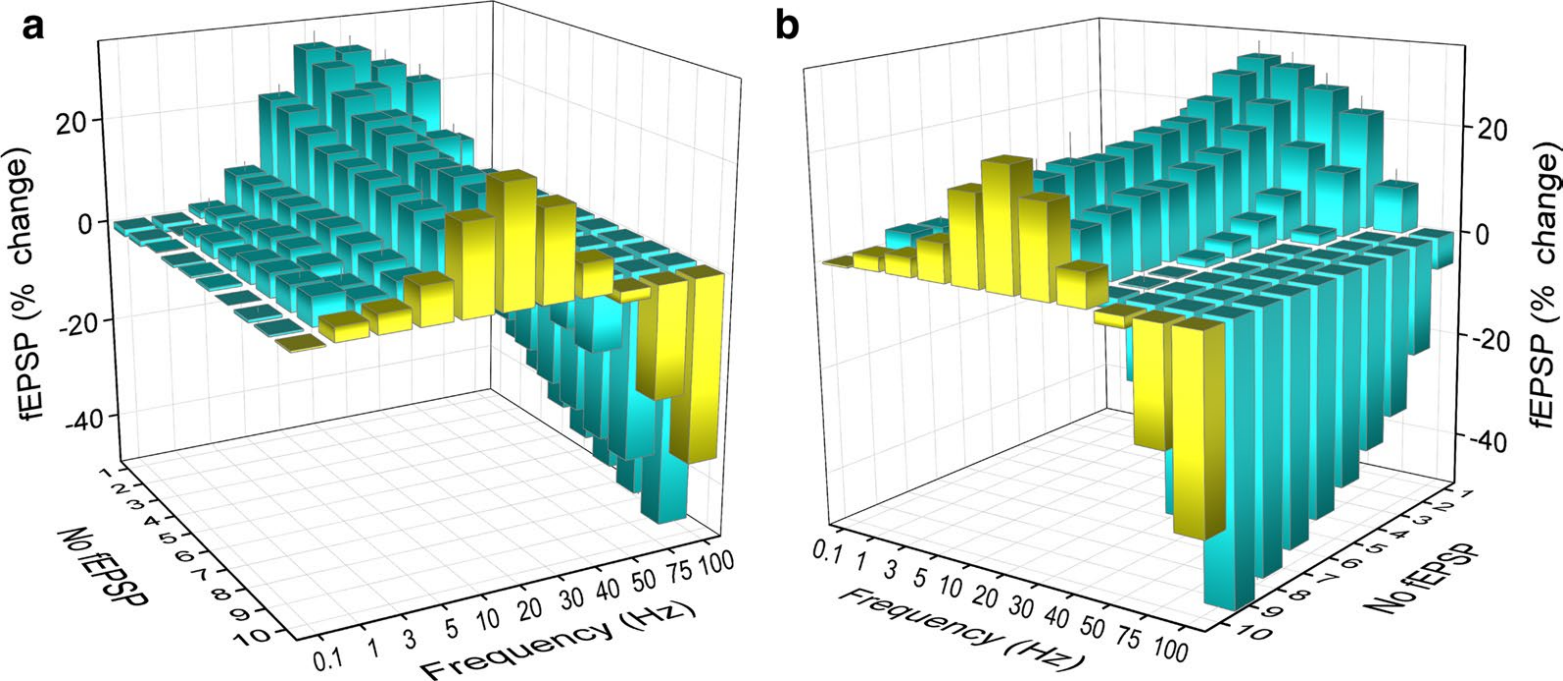
Averaged data of fEPSP changes obtained from the whole hippocampus (i.e. all slice positions) plotted against stimulus frequency and stimulus number. The left and right views of the graph are shown separately in panel **a** and **b** respectively. Note that, as a whole, hippocampal synapses optimally facilitate at frequencies of 10–30 Hz but they preferentially depress at 75–100 Hz. The set of data in yellow is the averaged values of fEPSP changes from the whole hippocampus and stimulation train across frequencies. Note that the hippocampal network shows facilitation between 1 and 50 Hz that peaks at 10–30 Hz and depression at 75–100 Hz

 These data suggest that the segment of the hippocampus that lies between the dorsal and the ventral poles of the structure is a place of transition of short-term synaptic plasticity properties. For instance, dorsal synapses in slices obtained from positions 2–6 and ventral synapses from positions 15–19 in the dorsoventral axis mainly facilitated and depressed respectively while synapses in intermediate hippocampus positions showed a comparable amount of facilitation and depression depending on the stimulus frequency and the number of stimuli. Thus, we decided to examine and compare the synaptic responses elicited in the three distinct hippocampal segments. Accordingly, we divided the dorsoventral axis of the hippocampus into three segments, the dorsal, the medial and the ventral hippocampus that corresponded to positions 2–7, 8–14 and 15–19 along the dorsoventral axis respectively. This division was based on the consistency of facilitated response displayed by the most dorsal synapses, the fact that both the steady-state response and the average of all conditioned responses elicited with medial range stimulus frequency (10–30 Hz) changes from facilitation to depression between the 14th and 15th position along the dorsoventral axis (Fig. 4) as well as on previous observations that have shown that the paired-pulse facilitation of the late phase of fEPSP drops abruptly after the 14th slice position along the dorsoventral hippocampus axis [37]. As anticipated, the three hippocampal segments displayed distinct synaptic behavior at almost all stimulus frequencies from 1 to 100 Hz and the pattern of synaptic responses changed gradually from the dorsal to the ventral hippocampus (Fig. 6). In particular, dorsal synapses typically facilitated from 1 to 50 Hz. At stimulus frequencies 1-3 Hz the facilitation significantly increased during stimulation (ANOVA, F = 3.1 for 1 Hz and 3.2 for 3 Hz, *P* < 0.005), while at frequencies 5-30 Hz the facilitation was stable during stimulation (ANOVA, 0.1 ≤ F ≤ 1.9, *P* > 0.05). Maximum facilitation in dorsal hippocampal slices was evoked at 20 Hz. At 75 and 100 Hz dorsal synapses transiently facilitated during the first 1–2 pulse and then increasingly depressed (ANOVA, 75 Hz, F = 32.9 and 100 Hz, 33.2; *P* < 0.001). On the contrary, ventral hippocampal synapses depressed from 1 to 100 Hz over the greater part of stimulation train showing only a transient and moderate facilitation at the start of 10–50 Hz stimulus trains. The depression significantly increased with stimulus progression at all frequencies ≥ 10 Hz (ANOVA, 2.9 ≤ F ≤ 16.0, *P* < 0.005). The synaptic responses in the medial hippocampus were typically intermediate between those of the dorsal and ventral hippocampal synapses for most stimulus frequencies. A commonality of all hippocampal segments was the strong depression of steady-state responses evoked at high stimulus frequencies (75–100 Hz). Stimulation at 0.1 Hz did not produce any significant change in synaptic transmission in any hippocampal segment. It should also be noted that the general pattern of dorsal hippocampal synaptic responses is similar to that previously observed in dorsal hippocampal slices [42]. Finally, when the synapses along the hippocampus long axis are treated as a uniform entity showed facilitation from 1 to 40 Hz that maximized at 20 Hz and for frequencies greater than 50 Hz it shifted to increasing depression (Fig. 7).

## Discussion

The present evidence demonstrates that Schaffer collateral synapses display strikingly different frequency-dependent dynamics along the dorsoventral axis of the hippocampus following a ten-pulse stimulus train. The main findings are as follows: (a) synapses at most dorsal locations in the hippocampus facilitate across a wide range of stimulus frequencies (1–50 Hz) showing maximal facilitation at 20 Hz, while at higher frequencies (75–100 Hz) they increasingly depress; (b) the steady-state of synapses at most ventral locations is depression over the entire range of stimulus frequencies (1–100 Hz); yet, ventral synapses showed a transient facilitation at the onset of 10–50 Hz stimulation; (c) synapses at the medial segment of the hippocampus show intermediate dynamics, thus revealing that the specialization in short-term synaptic plasticity is gradual along the dorsoventral axis of the hippocampus. These results demonstrate that there is a frequency-dependent gating of information at CA3-to-CA1 synapses that changes gradually along the dorsoventral hippocampal axis and strongly suggest that synaptic information processing is gradually diversified accordingly. Furthermore, they corroborate previous evidence showing that the local neuronal circuitry is specialized along successive segments along the longitudinal axis of the hippocampus.

### Possible interpretations of results

The reliability of a presynaptic spike to produce a postsynaptic response depends on the basal probability of transmitter release from the presynaptic terminal [45]. Furthermore, the probability of release is a function of the temporal structure of presynaptic action potentials [46, 47], thereby determining the pattern of postsynaptic responses upon repetitive presynaptic activity. Whether a synapse facilitates or depresses largely depends on its basal transmitter release probability and typically, synapses with a low initial transmitter release probability tend to facilitate while synapses with a high release probability tend to suppress [23, 34].

Previous observations [20, 35–38, 48] have suggested that the dorsal and the ventral hippocampal synapses have a low and high probability of transmitter release respectively [35, 37]. Accordingly, the varying pattern of synaptic responses along the hippocampus long axis, to some extent, may be accounted for by the differences in basal probability of transmitter release. Yet, the postsynaptic response to a presynaptic train of spikes depends on additional presynaptic mechanisms [23, 27, 31, 42, 47, 49]. For instance, a previous study has shown that the transmission at the CA3 to CA1 synapses during repetitive presynaptic activation, in addition to the basal transmitter release probability, depends on the relative contribution of two opposite presynaptic calcium-dependent processes, namely the facilitation of transmitter release induced during repeated activation and the recovery from refractory depression (facilitation-depression model) [42].

Strikingly, our results on the frequency-dependent synaptic responses at different segments of the hippocampus long axis are very similar to those predicted by the facilitation-depression model under conditions of presence or absence of these two processes [42]. According to this model, in the presence of both facilitation and recovery from depression at the dorsal CA1 synapses the release probability increases and the rate of recovery from depression accelerates at stimulus frequencies between 5 and 20 Hz leading to facilitation. Also, the steady-state release is maximal at a stimulus frequency of ~ 12 Hz. At relatively high stimulus frequencies (> 50 Hz) synaptic depression prevails because of deficient recovery. Similarly, we found that dorsal hippocampal synapses increasingly facilitate for frequencies up to 20 Hz, while they depress at frequencies > 50 Hz. On the other hand, the behavior of ventral hippocampal synapses can most accurately be explained by the absence of both processes, i.e. facilitation and recovery from depression, while the responses in the medial hippocampus are similar to those predicted by the presence of facilitation only [42]. It is therefore reasonable to assume that the gradual transition from facilitating dorsal synapses to depressing ventral synapses observed in the present study may probably be accounted for by a different relative contribution of facilitation and recovery from depression along the hippocampus long axis. Lastly, the inability of most hippocampal synapses to show significant change at the steady-state for low stimulus frequencies (0.1 Hz) is consistent with the absence of significant influence of either facilitation or refractory depression, because at low activity rates the accumulation of calcium at the presynaptic terminal is insufficient to mobilize these two processes [42].

Furthermore, additional mechanisms may contribute to the different synaptic properties observed over different frequencies and along the long hippocampal axis. In particular, synaptic inhibition may play an important role at relatively high stimulus frequencies, above 40–50 Hz, where depression prevails over facilitation. A previous study has shown that during activation at 50–100 Hz there is an increased activity of specific interneurons in CA1 that mediate strong inhibition of apical pyramidal cell dendrites [50]. Thus, under conditions of high-frequency stimulation strong inhibition of CA1 pyramidal cell apical dendrites may prevail over facilitation. Interestingly, the inhibition of dendrites increases from the first 3–4 stimuli [50], thereby presumably contributing to increasing frequency depression thatwe found in ventral-medial and dorsal hippocampus, at frequencies > 40 Hz and 75–100 Hz respectively. The fact that the frequency depression was stronger in ventral than in dorsal hippocampus may suggest that the activity of the specific type of interneurons that are recruited with high-frequency stimulation and mediate strong dendritic inhibition, which is presumably the oriens-lacunosum molecular interneuron [50], is stronger in the most ventral segment of the hippocampus. Interestingly, the number of GABAergic interneurons in stratum lacunosum moleculare is significantly higher in the ventral than in the dorsal hippocampal CA1 field [51].

### Possible general implications for information processing and activity flow in the hippocampus

Activity-dependent short-term plasticity endows synapses with frequency-filtering properties because transforms trains of presynaptic action potentials into postsynaptic responses of varying amplitude depending on the presynaptic firing frequency [26, 30, 52–56]. Short-term changes in synaptic effectiveness appear to play fundamental roles in neural information processing strongly influencing the network-level dynamics [11, 25, 26, 30, 31, 46, 49, 54, 57, 58]. It has been theorized that the various short-term dynamics can constitute a mechanism for the generation of a potentially unlimited diversity of synaptic inputs [59] endowing synapses with paramount computation abilities [26, 31, 53, 58, 60]. Importantly, different synapses in different cortical structures display very different synaptic dynamics [22, 42]. Facilitating synapses with a low basal probability of transmitter release transmit information of relatively increased presynaptic activity and therefore act as high-pass filters; depressing synapses with a high basal transmitter release probability that preferably transmit low frequency presynaptic activity function as low-pass filters, whereas synapses with intermediate release properties behave as band-pass filters [26]. Considering the different characteristics of short-term synaptic plasticity found in different segments along the longitudinal axis of the hippocampus it could be claimed that the synaptic dynamics in the hippocampal circuitry cover a wide range of distinct frequency-filtering properties, which are unevenly distributed along the long hippocampus axis.

Experimental and theoretical data shows that facilitating synapses more reliably code temporal information contained in relatively long trains of presynaptic spikes [26, 56]. On the other hand, depressing synapses are more suitable to transmitting significant information about the timing of up to four action potentials at the onset of presynaptic activity [26]. We found that dorsal hippocampal synapses steadily facilitate at all frequencies ≤ 50 Hz, while the ventral hippocampal synapses, which otherwise show dominant depression, only transiently facilitate at the beginning of the presynaptic stimulation train (i.e. the first 1–2 responses) delivered at frequencies between 10 and 50 Hz. Therefore, dorsal hippocampal synapses appear to be reliable for processing of continuously incoming information, whereas ventral hippocampal synapses seem to be adapted to signal information when there is a subtle but abrupt change in presynaptic activity. Furthermore, synapse in the medial segment of the hippocampus seems able to cover an intermediate range of information processing.

Among the most intriguing observations in the present study was that considerable steady state synaptic facilitation occurred in the dorsal and medial hippocampus at stimulus frequencies between ~ 3 and 40 Hz with a peak at 20 Hz. Hippocampus generates several behavior-related coherent network oscillations over distinct frequency domains including theta (4–10 Hz) beta (10–30 Hz) and gamma (~ 30–100 Hz) rhythms [61–64]. Thus, synaptic facilitation in the present study occurred at a frequency range extending from theta to low gamma with a peak at the beta frequency band. In particular, the fact that CA3-CA1 synapses most prominently facilitate at 10–30 Hz may suggest that activity propagation along the transverse and perhaps over a large portion along the dorsoventral axis of the hippocampus is favored when local network activity occurs at the beta frequency bands. Furthermore, because CA1 is the output of hippocampus to extrahippocampal regions this frequency range may be also used for optimal activity spread from hippocampus to other brain regions. Indeed, a recent study has shown that hippocampal output propagates from the hippocampal CA1 field towards several specific neocortical and subcortical areas preferentially at 10–20 Hz with a peak at 20 Hz [29]. It is also interesting that the abrupt decline in facilitation at stimulus frequency > 40 Hz observed here resembles the abrupt decline of extrahippocampal activity propagation at 40 Hz [29]. Moreover, substantial facilitation occurred also at a stimulus frequency of 5 Hz suggesting that activity at the theta frequency band significantly contributes to information spread in the transverse axis and along the dorsal-to-middle segment of hippocampus. Indeed, theta oscillation is used for activity propagation along the long hippocampus axis [65] in the dorsal–ventral direction [66]. It would also be relevant to note that the power of theta rhythm is higher in the dorsal than in the ventral hippocampus segment [67, 68]. Finally, because effective frequency-dependent gating of information flow appears to occur in the dorsal and medial segment but not the ventral hippocampus, the hippocampal output could more selectively be routed to other brain regions from the dorsal-to-medial hippocampus mainly at the beta frequency range.

### Possible implications for facilitating dorsal hippocampal synapses

One of the features of temporal filtering is burst detection [26]. Low probability facilitating synapses can accurately detect and effectively respond to short bursts of presynaptic action potentials [41], which is the typical mode of activity of pyramidal cells in the hippocampus [69]. By minimizing the number of active inputs that are required in order to trigger an action potential, presynaptic bursts can very reliably induce firing in the postsynaptic cell, thereby playing a critical role in the induction of long-term synaptic plasticity [41]. For instance, bursts of presynaptic stimuli patterned after the theta rhythm (i.e. 5 Hz) reduce the threshold for induction of long-term potentiation (LTP) (Larson-1986; Rose-Dunwiddie-1986). Importantly, dorsal but not ventral hippocampal synapses display strong facilitation and increased firing when presynaptic fibers are stimulated by theta bursts of electrical pulses [18] and bursting activity in presynaptic cells very effectively induces LTP in dorsal hippocampal synapses [44]. Increased synaptic facilitation and postsynaptic firing evoked by presynaptic bursts appears to underlie the mechanism by which dorsal hippocampal synapses display a lower threshold for LTP induction compared with ventral synapses [18]. Hence, by significantly assisting to induction of LTP, burst-evoked synaptic facilitation may provide dorsal hippocampus with increased ability to easily form memory traces of experienced events.

### Possible implications for depressing ventral hippocampal synapses

Synapses that display temporal dynamics in their transmission are crucial for the spontaneous generation of synchronous bursts in cortical structures [70]. Importantly, depressing synapses strongly increase the sensitivity of a neuron to small changes in the input firing rate [26, 30]. This may be particularly relevant for the initiation of the hippocampal network activity of sharp wave-ripples (SWRs), an endogenous activity of the hippocampus that plays important roles in memory consolidation [71]. SWRs requires subtle increases in the excitability of the local network [72] and are thought to be initiated from the most excitable pyramidal cells in the hippocampus [71], the activity of which can actually start from small changes in synaptic inputs [73]. Accordingly, the local ventral hippocampus circuitry with the increased sensitivity of its depressing synapses could be a favorable site of SWRs initiation. Indeed, in vitro SWRs are generated most likely in the ventral than in the dorsal hippocampus [74, 75].

Finally, depressing synapses in the ventral hippocampus may play a protective role against hyperexcitability. The ventral hippocampus is the most excitable segment of the hippocampus as exemplified by its increased susceptibility to epileptiform discharges, see ref. in [6]. Because increased excitability appears to be a basic property of the ventral hippocampus that supports physiological functions, there should be some intrinsic mechanisms that counterbalance the tendency of the ventral hippocampus for hyperexcitability. Theoretical and experimental work has shown that synaptic depression leads to rapid response saturation and reduces the response of local neural network to strong stimuli [49, 57] thereby preventing runaway synaptic enhancement [31, 49] that could otherwise drive local network into hyperexcitability. Hence, the depressing synapses in the ventral hippocampus may significantly contribute, among other mechanisms, to counteracting the endogenous tendency of this part of the hippocampus for hyperexcitability.

## Conclusions

Based on these results can be concluded that: (a) distinct short-term synaptic dynamics are segregated along the longitudinal axis of the hippocampus revealing a new aspect of hippocampal information processing and extending the concept of intrinsic diversification of the hippocampus; (b) the specialization in synaptic dynamics is gradual along the dorsoventral hippocampal axis permitting a wide range of different synaptic computations to be performed by the hippocampal neuronal network; (c) The specific characteristics of short-term synaptic plasticity may be crucially involved to diversify the functions along the hippocampus long axis. In particular, it is proposed that the gradual change in synaptic properties along the dorsoventral axis of the hippocampus endow it with a range of distinct capabilities, which can extend from an increased potentiality to undergo long-term synaptic modifications in the dorsal segment to an increased ability to initiate synchronized activities in the ventral segment of the structure. Finally, it is suggested that the flow of information in the CA3-CA1 pathway and presumably towards extrahippocampal targets is favored when neural activity is paced at the theta-beta frequency band.

## Abbreviations

ACSF: artificial cerebrospinal fluid
ANOVA: analysis of variance
CA1: cornu ammonis 1
CA3: cornu ammonis 3
fEPSP: field excitatory postsynaptic potentials
LTP: long-term potentiation
SWRs: sharp wave-ripples
